# From prevention to treatment of addiction: Porto’s comprehensive approach through the Municipal Health Plan

**DOI:** 10.1192/j.eurpsy.2025.836

**Published:** 2025-08-26

**Authors:** J. V. Santos, P. Pinto Oliveira, D. Lopes, A. Machado, S. Almeida, M. A. Moreira, E. Ramos, J. Coutinho, M. Pinto

**Affiliations:** 1 ULS Santo António / University of Porto; 2 ULS Santo António; 3 University of Porto / ISPUP; 4 CRI Porto Central; 5 University of Porto / CINTESIS, Porto, Portugal

## Abstract

**Introduction:**

The Porto Municipal Health Plan for 2022-2024 prioritized initiatives targeting addictions within the community. Built in the collaboration between the Public Health Unit, the Faculty of Psychology (FPCEUP), and the Drug Addiction Intervention and Reduction Division (DICAD), Porto aimed to comprehensively address addiction-related challenges. This includes developing monitoring tools, identifying areas lacking intervention, and promoting innovative social programs.

**Objectives:**

To define and implement a plan from prevention to treatment of addictive behaviors and addiction within the Porto Municipal Health Plan.

**Methods:**

After defining priorities within this scope, tasks were defined together with the different institutions of the Municipality, including disseminating information on tobacco and alcohol legislation and improving community literacy on health-conscious environments. Additionally, integrated projects focus on evaluating existing interventions, identifying best practices, and fostering collaboration among entities to address addiction effectively were listed as main steps.

**Results:**

By aligning with strategic objectives outlined in the Porto Municipal Health Plan, such as building citizen and professionals’ capacity, improving prevention strategies, and facilitating access to resources, Porto is addressing addictive behaviors comprehensively. Initiatives include capacity building, implementing intervention strategies, and promoting harm reduction approaches in recreational settings.

**Conclusions:**

Porto’s efforts to combat addiction highlight its commitment to public health. Through targeted communication, integrated projects, and resource optimization, Porto aims to mitigate the impact of addictive behaviors and promote a healthier community, aligning with the Municipal Health Plan.

**Disclosure of Interest:**

None Declared

